# The Differentiate Effects of Resistance Training With or Without External Load on Young Soccer Players’ Performance and Body Composition

**DOI:** 10.3389/fphys.2021.771684

**Published:** 2021-11-05

**Authors:** Moisés Falces-Prieto, Eduardo Sáez de Villarreal-Sáez, Javier Raya-González, Francisco Tomás González-Fernández, Filipe Manuel Clemente, Georgian Badicu, Eugenia Murawska-Ciałowicz

**Affiliations:** ^1^Research Center High Performance Soccer, Marcet Academy, Barcelona, Spain; ^2^Department of Football and Science, Pablo de Olavide University, Seville, Spain; ^3^Faculty of Health Sciences, Universidad Isabel I, Burgos, Spain; ^4^Department of Physical Activity and Sport Sciences, Pontifical University of Comillas, CESAG, Palma, Spain; ^5^SER Research Group, Pontifical University of Comillas, CESAG, Palma, Spain; ^6^Escola Superior Desporto e Lazer, Instituto Politécnico de Viana do Castelo, RuaEscola Industrial e Comercial de Nun’Álvares, Viana do Castelo, Portugal; ^7^Instituto de Telecomunicações, Delegação da Covilhã, Lisbon, Portugal; ^8^Department of Physical Education and Special Motricity, University Transilvania of Brasov, Brasov, Romania; ^9^Department of Physiology and Biochemistry, University School of Physical Education, Wrocław, Poland

**Keywords:** strength, VO_2_ max, performance, football, lean mass

## Abstract

**Purpose:** The purpose of this study was to examine the effects of 15 weeks (2/week) of two different resistance training (RT) programs [the self-load group (SG) vs. the overload group (OG)] on selected measures of physical performance in young male soccer players.

**Methods:** The countermovement jump (CMJ), aerobic endurance (VO_2_ max), and body composition [body mass (BM), height (H), body fat percentage (% BF), and lean mass (LM)] were measured before and after the 15-week RT interventions. Subjects were randomized to treatments: 1. SG [age = 15.34 ± 1.34 years]; 2. OG [age = 16.28 ± 1.21 years].

**Results:** The level of significance set for the study (*p* ≤ 0.05). Within-group analysis did report significant differences in all variables for the SG (*p* = 0.008 to 0.001; ES = −0.33 to 1.41, small to large) as in the OG (*p* = 0.001; ES = 0.82 to 1.30, large). Between-groups analysis reported differences in CMJ (*F* = 4.32; *p* = 0.004) for the OG.

**Conclusion:** The main findings of this study indicated that RT with and without external load was effective in improving the measures of physical performance in young soccer players, with special attention to jumping ability, where the OG group was more effective. Furthermore, there is no interference to aerobic endurance. It is recommended that soccer coaches implement RT without external load in the early stages of training or in players with late maturation development and in those soccer clubs with limited material resources.

## Introduction

Resistance training is considered a key strategy to improve in-field soccer performance due to the proved relationship between the strength level and high-intensity actions (e.g., sprint or jump) (Swinton et al., 2014; Núñez et al., 2019). In fact, the importance of resistance training (RT) has been increased in soccer training last year due to the relevance of this in the periodization (Lesinski et al., 2016). Concerning young athletes, it has shown RT to be important in preadolescence, highlighting neural plasticity associated with prepubertal players that support muscular strength development in these years through gains in neuromuscular adaptations as intra- and intermuscular coordination (Peña-González et al., 2019). Although different RT methodologies have been used to improve physical performance in soccer, such as programs bases on traditional exercises (Spineti et al., 2016), eccentric-overload training (Suárez-Arrones et al., 2019), plyometric training (Haghighi et al., 2012; Falces-Prieto et al., 2021), ballistic exercises (Loturco et al., 2020), Olympic exercises (Hori et al., 2008), electrostimulation training (Billot et al., 2010), and a combination of different methods (Raya-González and Sánchez-Sánchez, 2018). Most of these methods need expensive materials and equipment that preclude its applicability for most athletes and thus its implementation in most soccer training facilities; so strength and conditioning coaches are advised to find valid, simple, and economic resources for this purpose (Raya-González et al., 2020). Many coaches and physical trainers have taken this into consideration, and, accordingly, with the literature and their needs, professionals on soccer often choose RT based on self-loading (own body mass) as an interesting method that could be massively implemented in soccer training programs, especially in young soccer players (Peña-González et al., 2019). In fact, the effects of this RT based on self-loading have been previously analyzed in primary education students (Conde-Corbitante, 2016), adolescent basketball players (Kosmatos et al., 2008), prepubertal athletes (Faigenbaum and Myer, 2010), and elderly people (Kanda et al., 2018). However, to the best of our knowledge, there is a lack of evidence on the use of this RT programs in soccer players. Therefore, this highlights the need for studies that consider the effect of RT based on self-loading on young soccer players.

It is well-known that soccer is an intermittent exercise and involves activities with different high intensities, such as change of direction, high-intensity running, sprinting, jumps, and among others (Sáez de Villarreal et al., 2015; Raya-González and Sánchez-Sánchez, 2018). In this sense and considering the specific requirements of soccer in muscular strength terms, especially in the lower body to perform previous types of high-intensity actions mentioned (Michailidis et al., 2013), it should be mentioned that athletes need familiarization and adaptation with strength work (Peña-González et al., 2019), mainly due to the enormous importance of technical execution and time required for the proper implementation (Blais and Trilles, 2006). On the contrary, RT based on self-loading has shown that it is easier to apply in practice (Ferrete et al., 2014; Peña-González et al., 2019; Falces-Prieto et al., 2020). In addition, this methodology is seen as being more flexible, cheaper, quicker, and easier to implement on the day-to-day basis (Falces-Prieto et al., 2020). Notwithstanding, this methodology requires a great effort and a level of technical execution; otherwise, movements can be made with less control during execution (Falces-Prieto et al., 2020).

A review of the literature reveals that acute RT based on self-loading improves strength performance (Vanderka et al., 2016; Marín-Pagán et al., 2020). Consequently, if RT based on self-loading were repeated, it would also produce acute and chronical physiological changes. Therefore, the improvement can be related to the chronic adaptations of RT over long periods of time (Sander et al., 2013; Ferrete et al., 2014; Di Giminiani and Visca, 2017; Suárez-Arrones et al., 2018; Peña-González et al., 2019). Therefore, the direction and the magnitude of these phyisiological changes are due to work stimulus required and not the instrument applied.

In this first empirical study of the effects of two different RT programs on selected measures of physical performance in young male soccer players, we take a exploratory aproach to the study of efficiency of RT based on self-loading.

Countermovement jump (CMJ) was a valid test to observe the adaptation in training (Di Giminiani and Visca, 2017). Traditionally, the CMJ test is a standard measure of lower body power (Liebermann and Katz, 2003). In addition, it has been demonstrated a relationship between RT and CMJ improvement in young soccer players (Quagliarella et al., 2011; Comfort et al., 2014) with overloads (Comfort et al., 2014; De Hoyo et al., 2016; Falces-Prieto et al., 2021) and self-loads (Falces-Prieto et al., 2020; Ferrete et al., 2014; Peña-González et al., 2019). It is for this reason that RT has become crucial for young and adult soccer players (Moran et al., 2017).

Regarding the relationship between maximum oxygen consumption (VO_2_ max) and performance in soccer (Ziogas et al., 2011), the improvement of this variable could be a key strategy in the individual physical conditioning (Silva et al., 2011). Soccer players with higher VO_2_ max values present greater activity in high-intensity actions and sprinting and have a better recovery between high-intensity efforts (Nobari et al., 2021a). In recent years, a series of field tests have been designed in the evaluation of the VO_2_ max (Sánchez-Oliva et al., 2014). One of the tests commonly used in young soccer players is the 30–15 Intermittent Fitness Test (30–15 IFT) (Buchheit, 2008). Although endurance training inhibits or interferes with the development of RT and *vice versa* (Hennessy and Watson, 1994), previous studies have reported substantial improvements in VO_2_ max after RT programs in young soccer players (Ferrete et al., 2014; Ruivo et al., 2016; Marzouki et al., 2021). Even so, there are few studies that have examined the impact and adaptations of RT over VO_2_ max in soccer (Grieco et al., 2012), and, therefore, future research is needed.

Findings regarding anthropometric characteristics and body composition (BC) are of crucial importance for complex sports games such as soccer (Suárez-Arrones et al., 2019; Gardasevic et al., 2020). In addition, nonoptimal BC may adversely influence football performance and the risk of injury (Suárez-Arrones et al., 2019). There are studies that reflect a strong relationship between BC [high levels of lean mass (LM) and low-fat mass (FM)] with vertical jump performance and repeated sprint ability in both elite and youth soccer players (Rebelo et al., 2013; Brocherie et al., 2014; Nobari et al., 2021b). Regarding RT and its effects on BC in young soccer players, reflected increases in LM (Pérez-Gómez et al., 2008; Suárez-Arrones et al., 2019) and decrease in FM % (Suárez-Arrones et al., 2019; Falces-Prieto et al., 2021) after RT. Therefore, knowing the effects of different RT programs seems essential for their effective application and, consequently, improving the BC of young players.

In sum, the current empirical study was conceived to examine the effects of two different RT programs [the self-load group (SG) vs. the overload group (OG)] on selected measures of physical performance (i.e., jumping, aerobic endurance, and body composition) in young male soccer players. On the basis of the previous research on RT based on self-loading, we hypothesized that OG would induce larger adaptations on some measures of physical performance compared with SG in young male soccer players.

## Materials and Methods

### Experimental Approach to the Problem

**FIGURE 1:**
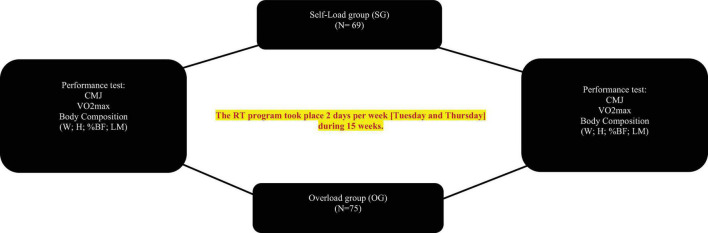
Schematic presentation of the study design. CMJ, countermovement jump; VO_2_ max, maximal oxygen consumption; U, under; RT, resistance training; SG, self-load group; OG, overload group; W, weight; H, height; % BF, % body fat; LM, lean mass.

To examine the effects of 15 weeks (2/week) of two different RTs [SG vs. OG] on selected measures of physical performance in young male soccer players [Under 16 (U16) and Under 19 (U19)] players participated in this study (Figure 1) were randomly assigned in two groups: SG (*n* = 69; soccer training program + RT program with self-load) and OG (*n* = 75; soccer training program + RT program with overload). Both groups were made up of players from both categories (U16 and U19). Before and after the RT, countermovement jump (CMJ), aerobic endurance (30–15 IFT) and body composition analysis (BC) [Weight (W, kg), height (H), body fat percentage (% BF), and lean mass (LM, kg) evaluated by bio impedance] were assessed.

### Participants

Initially, 150 young male soccer players belonging to the same high-performance academy agreed to participate in the study. The following inclusion criteria were applied to select subjects: (i) a background of ≥5 years of systematic soccer training and competitive experience, (ii) continuous soccer training for the previous 3 months with no musculoskeletal injuries, (iii) absence of potential medical problems, (iv) absence of any lower-extremity reconstructive surgery in the past 2 years, and (v) belongingness in the academy a full season. Subjects were required to attend ≥ 80% of all training sessions and attend all assessment sessions.

One hundred and forty-four young soccer players fulfilled the inclusion criteria and were randomly assigned to SG or OG. This study was conducted between the September and December of 2018/2019 season and consisted in a weekly resistance training session on Day 4 (Wednesday), allowing a rest of 72 h prior to a match and within the usual training hours (15:30–18:00 hours). The assessments were carried under weather conditions (∼ 29°C and ∼ 60% humidity) in September and (∼19°C and ∼ 50% humidity) in December. Only six subjects were excluded from the study because they were injured or were absent from the post-testing session. The subjects were randomized to treatments: 1. SG [age = 15.34 ± 1.34 years; height = 172.54 ± 7.18 cm; body mass = 62.69 ± 9.12 kg; % fat = 14.13 ± 3.78; lean mass = 53.85 ± 6.54 kg]; 2. OG [age = 16.28 ± 1.21 years; height = 174.18 ± 6.79 cm; body mass = 65.15 ± 8.21 kg; % body fat = 14.30 ± 3.52; lean mass = 56.10 ± 5.97 kg]. All participants were familiar with the training methods used and previous RT experience. Furthermore, completed 9 h of soccer training plus 1 competitive match per week. All parents and participants were informed about the purpose of the study and signed consent detailing their possible benefits and risks and giving the signed consent before the beginning of the study. The participants were fully debriefed about the purpose of the study at the end of the experiments. All players participated in 30 proposed sessions (100%). The participants were treated according to American Psychological Association (APA) guidelines, which ensured the anonymity of responses of the participants. In addition, the study was conducted in accordance with the ethical principles of the 1964 Helsinki declaration for human research and was approved by the Research Ethics Committee of the Pontifical University of Comillas (internal project No. 2021/65).

### Testing Procedures

#### Countermovement Jump Performance

The evaluation system was carried with a contact platform Chronojump-Boscosystem^®^ (Barcelona, Spain) (De Blas et al., 2012; Pueo et al., 2018). Three CMJ jumps were performed, with a recovery time of 20 s between jumps and the average of the three jumps for analysis (Falces-Prieto et al., 2021). The measurement was carried out with Chronopic and recorded with the Chronojump software version 1.4.7.0. Both for the pre- and post-evaluation of the CMJ, the subjects first performed a 10-min warm-up based on free joint and muscle mobility (3 min), skipping (2 × 30 s), gluteal heel (2 × 30 s), squats with extended arms (2 × 10 repetitions), and continuous vertical jumps (six jumps with the CMJ execution technique).

#### 1 RM Test

For the evaluation of the 1 RM for bench press and squat in OG and the subsequent programming of the training with these values, a linear encoder ChronojumpBoscoSystem^®^ (Barcelona, Spain) was used. Is an isoinertial dynamometer that consists of a cable extension linear position transducer attached to the barbell interfaced with a personal computer at a sampling rate of 1,000 Hz (Pérez-Castilla et al., 2019). It was measured to schedule training, but the effects on this variable were not assessed.

#### Aerobic Endurance

The 30–15 IFT, which consists of 30-s shuttle runs, interspersed with 15-s passive recovery periods. Velocity was set at 8 km/h^–1^ for the first 30-s run and was increased by 0.5 km/h^–1^ every 45-s stage thereafter (Buchheit, 2008). The test methodology served as a progressive warm-up of the test. The subjects had to run back and forth between two lines set 40 m apart at a pace governed by a prerecorded beep at appropriate intervals that helped them adjust their running speed by entering into 3-m zones at each extremity and in the middle of the field while the short beep sounds. It was established that the subject should stop the test when, for three consecutive times, he or she does not reach the established line at the rhythm of the prerecorded sound. When the subjects could not follow the speed stipulated in the test, they should raise their hands to signal their cessation and thus note the previous speed at which the player stopped. For the estimate of VO_2_ max, the following formula has been used (Buchheit, 2008):


V⁢O⁢m2⁢a⁢x=28.3-(2.15×G)-(0.741×A)-(0.0357×W)

+(.0586×A×v⁢I⁢F⁢T)+(1.03×v⁢I⁢F⁢T).

Variables: G: Gender (one man; two women); A: age; W: weight; vIFT: final speed reached.

#### Body Composition

Anthropometric measurements were taken before the physical testing. The stature of soccer players was measured with a stadiometer (Seca^®^ 206, Hamburg, Germany). The BC was evaluated in the morning (8:00 am) at the beginning of the competitive period (September) and at the end of the treatments (December). The variables BM, % BF, and LM were analyzed with the Bioelectrical Impedance Analysis method (BIA) using a TANITA^®^ (MC-980MA PLUS, Arlington Heights, IL, United States), where the subjects go up without footwear, without breakfast and wore only shorts and removed any metal and jewelry prior to assessment (Suárez-Arrones et al., 2019). BIA is a widely used method for estimating LM (Sun et al., 2005; Böhm and Heitmann, 2013) and offers a method that is economic and noninvasively assesses the fluid distribution and BC of young soccer players (Lozano-Berges et al., 2017).

#### Training Program

The subjects completed an ST program for 15 weeks. Cross the season, players had five to six training sessions a week, with an average duration of 80 min (from 45-min sessions to 100- to 120-min sessions where the ST training sessions were included before field training). During the intervention (15 weeks), the subjects performed five normal training sessions (soccer-specific trainings in the field) plus two RT sessions per week. In both groups, Day 1 was for the upper body and Day 2 for the lower body. It was carried out in a training circuit format. In SG, the intensity used was the body weight or body weight plus light resistance of the player (Ferrete et al., 2014; Peña-González et al., 2019). The training was performed on the artificial grass (the same as competition), with the subjects using appropriated soccer-equipped boots and clothes. Sets (4) and established repetitions (×12 × 10 × 8 × 8) were established. The OG performed RT in gym with overloads. The external overloads for the bench press and squat exercise were between 50 and 65% of the 1RM (Rodríguez-Rosell et al., 2017). The weight of the bar was taken into account (it was not Olympic; 11 kg). With respect to the rest of the exercises with overloads, the subjects used free weight by means of which they could complete the sets and prescribed repetitions and with the correct execution technique (Peña et al., 2016). According to the exercise to be performed, sets (4) and repetitions (×15 × 12 × 10 × 8 × 8) were established, with maximum execution speed. The resting period between each set in both treatments was 1 min. The RT program followed by the groups is outlined in Tables 1, 2.

**TABLE 1:** Phase 1(A): Self-load treatment.

**Weeks**	**W1**		**W2**		**W3**		**W4**		**W5**		**W6**		**W7**		**W8**	
**Exercises/Sessions**	**S1**	**S2**	**S3**	**S4**	**S5**	**S6**	**S7**	**S8**	**S9**	**S10**	**S11**	**S12**	**S13**	**S14**	**S15**	**S16**

**Day 1 upper body** – **Day 2 lower body**																
Front shoulders with EB	4×12		4×12		4×12		4×12		4×12		4×12		4×12		4×12	
Lateral shoulders with EB	4×12		4×12		4×12		4×12		4×12		4×12		4×12		4×12	
Normal Push-Ups	4×10		4×10		4×10		4×10		4×10		4×10		4×10		4×10	
Triceps Dips	4×8		4×8		4×8		4×8		4×8		4×8		4×8		4×8	
Biceps with EB	4×10		4×10		4×10		4×10		4×10		4×10		4×10		4×10	
Row with EB	4×12		4×12		4×12		4×12		4×12		4×12		4×12		4×12	

Squat with pica		4×12		4×12		4×12		4×12		4×12		4×12		4×12		4×12
Bipodal glute bridge		4×8		4×8		4×8		4×8		4×8		4×8		4×8		4×8
Calf lift		4×12		4×12		4×12		4×12		4×12		4×12		4×12		4×12
Quadriceps isometric 90°		4×30′′		4×30′′		4×30′′		4×30′′		4×30′′		4×30′′		4×30′′		4×30′′
Static Lunges		4×12		4×12		4×12		4×12		4×12		4×12		4×12		4×12
Monster Walk		4×8		4×8		4×8		4×8		4×8		4×8		4×8		4×8

*EB, elastic band.*

**TABLE 1:** Phase 1(B): Self-load treatment.

**Weeks**	**W9**		**W10**		**W11**		**W12**		**W13**		**W14**		**W15**	
**Exercises/Sessions**	**S17**	**S18**	**S19**	**S20**	**S21**	**S22**	**S23**	**S24**	**S25**	**S26**	**S27**	**S28**	**S29**	**S30**

**Day 1 upper body** – **Day 2 lower body**														
Decline push up	4×12		4×12		4×12		4×12		4×12		4×12		4×12	
Chest TRX	4×12		4×12		4×12		4×12		4×12		4×12		4×12	
Triceps TRX	4×8		4×8		4×8		4×8		4×8		4×8		4×8	
Row TRX	4×10		4×10		4×10		4×10		4×10		4×10		4×10	
Biceps TRX	4×8		4×8		4×8		4×8		4×8		4×8		4×8	
Throw medicine ball (4 Kg)	4×10		4×10		4×10		4×10		4×10		4×10		4×10	

Bipodal Squat TRX		4×8		4×8		4×8		4×8		4×8		4×8		4×8
Unipodal Squat TRX		4×8		4×8		4×8		4×8		4×8		4×8		4×8
Hamstrings TRX		4×10		4×10		4×10		4×10		4×10		4×10		4×10
Squat Quádriceps with strap		4x12		4×12		4×12		4×12		4×12		4×12		4×12
Hamstrings with strap		4×10		4×10		4×10		4×10		4×10		4×10		4x10
Nordic Hamstring		4×8		4×8		4×8		4×8		4×8		4×8		4×8
Hamstring kick with EB		4x8		4×8		4×8		4×8		4×8		4×8		4×8

*EB, elastic band; Kg, kilograms.*

**TABLE 2:** Phase 1(A): Overload treatment.

**Weeks**	**W1**		**W2**		**W3**		**W4**		**W5**		**W6**		**W7**		**W8**	
**Exercises/Sessions**	**S1**	**S2**	**S3**	**S4**	**S5**	**S6**	**S7**	**S8**	**S9**	**S10**	**S11**	**S12**	**S13**	**S14**	**S15**	**S16**

**Day 1 upper body** – **Day 2 lower body**																
Bench Press	4×15 (BW)		4×15 (BW)		4×15 50% RM		4×15 50% RM		4×15 50% RM		4×15 55% RM		4×15 55% RM		4×15 55% RM	
Curl Biceps	4×10		4×10		4×10		4×10		4×10		4×8		4×8		4×8	
Triceps Pulley	4×8		4×8		4×8		4×8		4×8		4×8		4×10		4×10	
Shoulders 30°	4x8		4×8		4×8		4×8		4×8		4×8		4×8		4×8	
Unilateral Row Machine	4×10		4×10		4×10		4×10		4×10		4×10		4×10		4×10	

Squat		4×15 (BW)		4×15 (BW)		4×15 50% RM		4×15 50% RM		4×15 50% RM		4×15 55% RM		4×15 55% RM		4×15 55% RM
Leg Curl		4×10		4×10		4×10		4×10		4×10		4×8		4×8		4×8
Hip Thrust		4×10		4×10		4×10		4×10		4×10		4×10		4×10		4×10
Adductor machine		4×10		4×10		4×10		4×10		4×10		4×10		4×10		4×10
Gluteus medius machine		4×10		4×10		4×10		4×10		4×10		4×10		4×10		4×10

*BW, bar weight (11 kg); Kg, kilograms; RM, repetition maximum.*

**TABLE 2:** Phase 1(B): Overload treatment.

*Weeks*	**W9**		**W10**		**W11**		**W12**		**W13**		**W14**		**W15**	
***Exercises/Sessions***	**S17**	**S18**	**S19**	**S20**	**S21**	**S22**	**S23**	**S24**	**S25**	**S26**	**S27**	**S28**	**S29**	**S30**

**Day 1 upper body** – **Day 2 lower body**														
Bench Press	4×15 55% RM		4×15 60% RM		4×15 60% RM		4×15 60% RM		4×15 60% RM		4×12 65% RM		4×12 65% RM	
Concentric curl	4×12		4×12		4×12		4×10		4×10		4×10		4×10	
Dumbbell triceps extension	4×8		4×8		4×8		4×8		4×8		4×8		4×8	
Press militar	4×8		4×8		4×8		4×8		4×8		4×8		4×8	
Assisted chin-ups	4×10		4×10		4×10		4×10		4×10		4×10		4×10	

Unipodal Squat		4×15 55% RM		4×15 60% RM		4×15 60% RM		4×15 60% RM		4×15 60% RM		4×12 65% RM		4×12 65% RM
Flexo-extensión fitball unipodal		4×12		4×12		4×12		4×12		4×12		4×12		4×12
Hip thrust		4×12		4×12		4×12		4×10		4×10		4×10		4×10
Eccentric adductors		4×12		4×12		4×12		4×12		4×12		4×12		4×12
Gluteus medius machine		4×10		4×10		4×10		4×10		4×10		4×10		4×10

*RM, repetition maximum.*

#### Statistical Procedures

Data are presented as mean ± standard deviations (SD). The ICC was used to determine the reliability of the measurements. To prove the normality of data distribution and the homogeneity of variances, the Kolmogorov-Smirnov and Levene tests were conducted. Since all analyzed variables had a normal distribution, parametric techniques were applied. A paired-samples t-test was used to evaluate within-group differences, and an analysis of covariance (ANCOVA) was performed to detect possible between-group differences, assuming baseline values as covariates. To examine practical significance, Cohen’s effect size was calculated (Cohen, 1988), and the obtained results were interpreted as follows: trivial (lower than 0.2), small (between 0.2 and 0.5), moderate (between 0.5 and 0.8), and large (above 0.8). These data were analyzed using the Statistical Package for Social Sciences (SPSS 25.0, SPSS Inc., Chicago, IL, United States), and the statistical significance was set at (*p* < 0.05).

## Results

In Table 3 are the presented changes in physical performance for both groups after the intervention period. Within-group analysis did report significant differences in all variables for the SG (*p* = 0.008 to 0.001; ES = −0.33 to 1.41, small to large) as in the OG (*p* = 0.001; ES = 0.82 to 1.30, large). Between-groups analysis reported differences in CMJ (*F* = 4.32; *p* = 0.004) for the OG.

**TABLE 3:** Physical performance before (baseline) and after (post-training) the 15-week intervention period in both groups.

	**SG (*n* = 69)**					**OG (*n* = 75)**					**Between-group differences**	
**Variable**	**Baseline Mean ± SD**	**Post training Mean ± SD**	**Δ (%)**	***p***	**ES**	**Baseline Mean ± SD**	**Post training Mean ± SD**	**Δ (%)**	***p***	**ES**	***F***	***p***
CMJ (cm)	31.355.24	33.694.93	7.46	**0.001**	1.22	31.153.76	34.244.10	9.91	**0.001**	1.30	4.32	**0.004**
VO_2max_ (ml/k/min)	49.013.13	50.742.67	3.53	**0.001**	0.85	48.672.92	50.182.86	3.10	**0.001**	0.82	1.20	0.275
Weight (kg)	62.389.01	64.638.46	3.61	**0.001**	0.82	64.518.33	66.757.73	3.47	**0.001**	0.91	0.30	0.586
Height (cm)	172.577.40	173.577.21	0.58	**0.001**	0.91	173.376.65	174.086.56	0.41	**0.001**	0.93	1.68	0.095
Body fat (%)	13.353.01	12.892.66	–3.45	**0.008**	–0.33	14.613.71	13.643.18	–6.64	**0.001**	–0.95	2.37	0.126
Lean mass (kg)	53.886.99	55.996.49	3.92	**0.001**	1.41	55.525.73	57.155.59	2.94	**0.001**	1.30	2.76	0.099

*SG, self-load group; OG, overload group; SD, standard deviation; Δ (%), percentage of change between pre and post conditions; *p*, a level of significance (*p* ≤ 0.05); ES, effect size; CMJ, countermovement jump.*

## Discussion

The aim of this study was to examine the effects of two different RT programs [the self-load group (SG) vs. the overload group (OG)] in physical performance of young male soccer players. The main findings of this study indicated that RT with and without external load was effective in improving jumping, aerobic endurance, and body composition in young soccer players, with special attention to jumping ability, where the OG group was more effective. To our knowledge, it is the first research that compares the two RT methods for young soccer players and shows a general benefit in the variables evaluated for young soccer players and could help professionals and coaches of preadult soccer players.

The demands of physical performance in soccer are related to actions of maximum and explosive strength (Sáez de Villarreal et al., 2015). In terms of CMJ performance, our findings showed significant performance improvements for the CMJ in both groups [SG: *p* < 0.001; OG: *p* < 0.001)] most effectively for the OG (*p* < 0.004). Little research had been conducted regarding the effects of RT without external load compared with external load in CMJ performance in male soccer players; therefore, current results are difficult to discuss. Regarding the use of RT without external loads, our results in SG (*p* < 0.001) after 15 weeks, coincide with different studies. Ferrete et al. (2014) trained 2 days a week for 26 weeks in young soccer players using the body weight of the player (or body weight plus light resistance) as external resistance and found improvements in CMJ in the experimental group (*p* ≤ 0.05). Falces-Prieto et al. (2020) also showed improvements in CMJ (*p* < 0.01) in U19 soccer players after performing RT with self-loading for 2 days a week for 8 weeks. In accordance with our results in OG (*p* < 0.001) after 15 weeks, previous studies have also reported similar increases in jump ability (Franco-Márquez et al., 2015; Rodríguez-Rosell et al., 2017) after RT programs with similar duration (6–8 weeks) load (45–60% 1 RM) and training frequency (2 days per week) among young soccer players. Therefore, our results reinforce the validity of both RT methods in young soccer players to improve jumping ability.

The published training programs advise soccer players to simultaneously train strength and endurance qualities, since they are two of the most important physical abilities to develop in soccer (Hennessy and Watson, 1994). Therefore, the combination of both qualities can activate different anabolic or catabolic processes that are modulated by endocrine responses to exercise and training, producing positive adaptations in the body (Sporiš et al., 2011). That is why the results obtained in this study (*p* < 0.001) in both groups are in line with those of the researchers who justify the positive effect of RT on endurance capacity in young soccer players (Sáez de Villarreal et al., 2015; Ruivo et al., 2016; Di Giminiani and Visca, 2017). Ferrete et al. (2014) also evidenced significant increases (*p* ≤ 0.05) in the VO_2_ max after RT for 26 weeks using the body weight of the player (or body weight plus light resistance) as external resistance in young soccer players. Regarding the RT with external load, Ruivo et al. (2016) showed improvements in VO_2_ max (*p* ≤ 0.05) in young soccer players after RT (∼ 65% 1 RM; 3 days a week; 16 weeks). It should be noted that there are few studies that have examined the impact and adaptations of both positive and negative RTs over endurance capacity in young soccer players; we cannot reinforce our data, and, therefore, future research is necessary (Grieco et al., 2012). However, our results can be considered advantageous in young soccer players, because it is observed that, after RT, there is no interference of aerobic endurance.

The BC of young people undergoes rapid changes during their growth spurts, with substantial changes in H and W (Suárez-Arrones et al., 2019). Also interesting is the fact that young soccer players show a high percentage of BF due to absolute low levels of LM and not high levels of BF *per se* (Suárez-Arrones et al., 2019) so that our results show that both ST treatments have been shown to be effective in improving the BC parameters evaluated (SG: *p* < 0.001; OG: *p* < 0.001). Our data on W are in accordance with the study of Erdem-Cigerci and Genc (2020), which examined the effect of calisthenics strength exercises performed 3 days a week for 8 weeks and found significant W increases (*p* < 0.032) in the experimental group. Ferrete et al. (2014) also found significant increases in W (*p* ≤ 0.05) after performing 2-day RT a week for 26 weeks in young soccer players using the body weight of the player (or body weight plus light resistance) as external resistance. Regarding the RT with external load, Ruivo et al. (2016) showed significant increase in W (*p* ≤ 0.05) in young soccer players after RT.

There is a popular belief that RT when an individual has not yet fully developed negatively affects his or her growth or modifies his or her final H (Faigenbaum et al., 2009; Faigenbaum and Myer, 2010). However, no scientific evidence has been found on growth in young athletes who performed RT programs under qualified supervision and appropriate prescription (Faigenbaum et al., 2009; Faigenbaum and Myer, 2010; Peña et al., 2016). Regarding the improvement in the H variable with both treatments, our data are in accordance with the study by Sander et al. (2013), who evaluated the influence of an RT program for 2 years in young soccer players (*n* = 134) divided into three age groups (A: 17 years; B: 15 years; C: 13 years), and they also found significant improvements in growth in the three categories (*p* < 0.05). Ferrete et al. (2014) also showed significant increases in the H (*p* ≤ 0.05) in young soccer players.

Another benefit of the RT is associated with the lowering of the % BF, which can be beneficial for a football player (Ruivo et al., 2016). We can indicate that treatments without external overload (*p* < 0.008) and with external overload (*p* < 0.001) have been shown to be effective for the decrease of % BF. Equally, Falces-Prieto et al. (2020) also showed decreases in the % BF in U17 and U19 (*p* < 0.001) young soccer players after performing RT with self-loading during 8 weeks. In addition, Suárez-Arrones et al. (2019) showed a significant decrease in % BF (ES = −0.99) after the RT program (i, RT in the gym combining free weights with flywheel inertial devices; ii, specific RT on the field; iii, individual training) organized as circuit training in young soccer players during 26 weeks. Finally, with respect to LM, both RT treatments showed significant improvements (*p* < 0.001), confirming our hypothesis. This coincides with the proposed by Milsom et al. (2015), which suggested that training should be more focused on the gain of LM and not the reduction of BM. In addition, having high LM levels allows the player to avoid traumatic injuries derived from contact and a decrease in the probabilities of muscle injuries (Keiner et al., 2014; Perroni et al., 2015). Our results are in agreement with Pérez-Gómez et al. (2008), where they analyzed the effects of an RT program, consisting of weight lifting, combined with plyometric exercises, followed a period of 6 weeks with 3 sessions/week in U16 soccer players, with an increase in significance in LM (*p* ≤ 0.05). We can indicate that the benefits of the treatments proposed in this study are similar to the results obtained with other treatments such as eccentric overload (Suárez-Arrones et al., 2018) and self-loading (Falces-Prieto et al., 2020) among others in young soccer players.

It has been clearly shown that young soccer players can improve jumping, aerobic endurance, and body composition through two different RT programs (with and without external loads) performed 2 days a week for 15 weeks during the season. Furthermore, there is no apparent interference between the development of RT and the other qualities evaluated. Such benefits can be realized from only two RT training sessions per week in season. The performance improvements shown in this study are of great interest for soccer coaches and are directly applicable to prepubertal, young, and professional soccer players. In addition, the present study confirms that the RTs without and with external loads are some valid methods to produce changes at the neuromuscular, cardiovascular level and modification of BC in young soccer players. Previous authors have found similar benefits of RT in this sport, but this is the first study to our knowledge that proposes a self-loading RT methodology and its benefits on different physical qualities on young soccer players. Therefore, it should be considered during the prescription of RT by coaches and fitness coaches of soccer. The outcomes may help soccer coaches and sport scientists formulate better guidelines and recommendations for assessment and selection, training prescription and monitoring, and preparation for competition of young soccer players.

This study had some limitations. One of the main limitations was the absence of a control group, which is mainly needed to isolate the effects of resistance training from those due to growth and maturation. Second limitation was the control of the soccer-specific training loads. The subjects attended their usual soccer training with their usual teams, and differences between the soccer-specific training loads may appear. Third was that nutritional parameters were not taken into account. Nevertheless, the findings of the present research are an important strength. In fact, the intervention applied for 15 weeks (2/week) represents an innovative line of work that coaches should be considerate in their training seasons planning. Future studies may analyze if the load control and nutritional advice may induce more favorable effects. Irrespective of this, the methodology used in this research can be a good initiation treatment to ST in young soccer players, being very useful. It is worth noting that the players reported positive feelings and enjoyment regarding the training intervention as well as the technical staff, indicating the desire to maintain the strength and conditioning program during the season.

## Conclusion

The findings of this study demonstrate that young soccer players can enhance muscle strength, aerobic endurance, and body composition by undertaking a 15-week in-season program with RT with and without external loads with special attention to training with external load is more effective to improve the jump ability. These results have no apparent interference between the development of RT and aerobic endurance and height in young soccer players. For this reason, the finding encountered in terms of performance suggests that RTs are crucial for young soccer players. Furthermore, this information could be useful for soccer coaches and technical staff due to its potential applicability to soccer performance. In fact, performance on soccer relies greatly on the specific on-field vertical jump, aerobic endurance, and high levels of lean mass. Previous authors have found a similar benefit of strength and power training in others and this sport, but this is the first study, to our knowledge, involving RT with and without external loads and its relationship with improvements in different performance parameters in soccer. It is recommended that soccer coaches implement strength training without external load in the early stages of training or in players with late maturation development and in those soccer clubs with limited material resources. The outcomes may help coaches and sport scientists formulate better guidelines and recommendations for athlete assessment and selection, training prescription and monitoring, and preparation for competition.

## Data Availability Statement

The raw data supporting the conclusions of this article will be made available by the authors, without undue reservation.

## Ethics Statement

All procedures were approved by the local ethics committee for the use of human participants in accordance with the latest version of the Declaration of Helsinki. Written informed consent to participate in this study was provided by the participants’ legal guardian/next of kin.

## Author Contributions

MF-P and ES led the project, established the protocol, drafted the initial manuscript, and reviewed and revised the manuscript. JR-G, FG-F, and FC wrote and revised the manuscript. GB and EM-C wrote and reviewed the final version. All authors approved the final manuscript as submitted and agree to be accountable for all aspects of the work.

## Conflict of Interest

The authors declare that the research was conducted in the absence of any commercial or financial relationships that could be construed as a potential conflict of interest.

## Publisher’s Note

All claims expressed in this article are solely those of the authors and do not necessarily represent those of their affiliated organizations, or those of the publisher, the editors and the reviewers. Any product that may be evaluated in this article, or claim that may be made by its manufacturer, is not guaranteed or endorsed by the publisher.
